# Time of day bias for biological sampling in studies of mammary cancer

**DOI:** 10.1038/s41598-023-50785-y

**Published:** 2024-01-08

**Authors:** James C. Walton, William H. Walker, Randy J. Nelson, A. Courtney DeVries

**Affiliations:** 1 Department of Neuroscience, Rockefeller Neuroscience Institute, West Virginia University, Morgantown, WV 26505 USA; 2 Department of Medicine, Division of Oncology/Hematology, West Virginia University, Morgantown, WV 26505 USA; 3 West Virginia University Cancer Institute, West Virginia University, Morgantown, WV 26505 USA

**Keywords:** Neuroimmunology, Breast cancer, Circadian rhythms and sleep, Neuroimmunology

## Abstract

Despite its demonstrated biological significance, time of day is a broadly overlooked biological variable in preclinical and clinical studies. How time of day affects the influence of peripheral tumors on central (brain) function remains unspecified. Thus, we tested the hypothesis that peripheral mammary cancer tumors alter the transcriptome of immune responses in the brain and that these responses vary based on time of day; we predicted that time of day sampling bias would alter the interpretation of the results. Brain tissues collected at mid dark and mid light from mammary tumor-bearing and vehicle injected mice were analyzed using the Nanostring nCounter immune panel. Peripheral mammary tumors significantly affected expression within the brain of over 100 unique genes of the 770 represented in the panel, and fewer than 25% of these genes were affected similarly across the day. Indeed, between 65 and 75% of GO biological processes represented by the differentially expressed genes were dependent upon time of day of sampling. The implications of time-of-day sampling bias in interpretation of research studies cannot be understated. We encourage considering time of day as a significant biological variable in studies and to appropriately control for it and clearly report time of day in findings.

## Introduction

Time of day is a critical biological variable that is largely overlooked in both preclinical and clinical study and treatment of diseases^1–3^. Most biological processes display circadian rhythmicity driven by internal circadian clocks organized in a hierarchical fashion at the cellular, organ, and organismal level. Disruption of rhythms or desynchrony in circadian rhythmicity at any of these levels tends to result in sub-optimal functioning and poor outcomes for individuals. Indeed, both susceptibility to disease and immune responses vary across the day^4,5^, and disruption of circadian rhythms exacerbates virtually all diseases studied in human and animal models of human diseases, spanning mental health to vascular diseases and cancer^6–9^.

Previous work has demonstrated reciprocal interactions between peripheral cancer and the brain^10–14^. Indeed, activation of the reward system of the brain can modulate anti-tumor immunity in mouse models of lung carcinoma and melanoma^13^. Stimulation of hypothalamic oxytocin neurons can suppress colorectal cancer progression in mice^15^. Futhermore, foundational studies have demonstrated the ability of peripheral mammary tumors to alter depressive-like behavior, cognitive function, and sleep^10–12^. Clinical studies support these findings as cancer patients commonly experience elevated depression and anxiety, as well as impaired cognitive function and sleep^16–20^. Whereas the precise underlying mechanism remains unspecified, the balance of empirical evidence supports the hypothesis that neuroinflammation is responsible for the neuropsychological and sleep disturbances observed in cancer patients.

Notably, time of day is a critical variable in tumor development, metastatic growth, and chemotherapeutic treatment^21–26^. However, very few studies have examined the effects that peripheral tumors can have on the immune transcriptome within the brain, and the changes in expression patterns across the day^27^. With these factors in mind, we tested the hypothesis that mammary cancer alters the transcriptome of immune responses in the brain and that these responses vary based on time of day.

## Methods

All procedures were approved by West Virginia University’s Institutional Animal Care and Use Committee and were performed in accordance with all the relevant guidelines and regulations, and the study is reported in accordance with ARRIVE guidelines. Thirty-two adult female Balb/C mice (8 weeks of age at arrival) were obtained from Charles River Labs (Wilmington MA USA). Upon arrival, mice were individually housed in polycarbonate cages, had ad libitum access to food (Teklad #2018) and RO-filtered tap water, and allowed to acclimate for a week to Light:Dark (LD) 14:10 cycles {lights on zeitgeber time (ZT) 0, 05:00 h; lights off ZT14, 19:00 h} prior to any manipulation. After acclimation, each mouse received an orthotopic injection into the 4th and 9th inguinal mammary glands as previously described^28^. Half of the mice (n = 16) were injected with mycoplasma-free murine non-metastatic 67NR cells (1.0 × 10^5^ cells in DMEM, volume 0.1 mL), the remainder (n = 16) were injected with vehicle only (DMEM 0.1 mL). Injections occurred from ZT5-ZT7 (i.e. 5–7 h after lights on). The 67NR cell line is a non-metastatic mammary carcinoma cell line that was isolated from a single spontaneous Balb/cfC3H mammary tumor and obtained from Barbara Ann Karmanos Cancer Institute (Detroit, MI)^29^. Following injection, mice were returned to their home cages for recovery. On the 25th day after injection, half of the mice in each group were euthanized for tissue collection at one of two timepoints; mid-dark (ZT19, n = 16), or mid-light (ZT7, n = 16). Mice were transcardially perfused with ice cold 1X phosphate-buffered saline and decapitated. Adequate perfusion was confirmed based on clearance of blood from the liver. To preserve RNA integrity, brains were rapidly collected into RNAlater (Thermo Fisher) and stored in − 80 °C overnight for later processing. 67NR-injected mice were verified to have mammary tumors, and vehicle injected mice were verified to be mammary tumor free.

After preservation in RNAlater, brains were dissected, and RNA was extracted from the hypothalamus (HYP), the hippocampus (HPC), and brainstem (BS) using Trizol according to manufacturer’s instructions. Samples were then coded to mask the groups and sent to the Johns Hopkins Genetic Resources Core Facility for further processing and analyses. RNA quality of each sample was verified using an Agilent 2100 Bioanalyzer (Agilent Technologies); verified samples (n = 5 from each experimental condition) were then assayed using the nanoString nCounter Mouse Neuroinflammation Panel to simultaneously assesses expression levels of 770 neuroinflammation-related genes (NanoString Technologies), among them is a subset of genes NanoString has annotated as immune cell-type markers^30^.

The purified mRNA first underwent quality control to determine concentration and RNA integrity. The samples were then processed following NanoString recommended protocol on their PrepStation and analyzed with their Digital Analyzer on nCounter cartridges for the nCounter Mouse Neuroinflammation Panel v1. Following Analyzer scanning the raw data were transferred and normalized with the NanoString nSolver Analysis v3 software platform, employing: Negative Background Subtraction, Positive Control Normalization, and CodeSet Content Normalization using geometric mean values. The processed expression values were then exported and further analyzed using the Partek GS v6.6 (Partek Inc.) platform, where they underwent quantile normalization. The different biological classes were then compared using the Student's *t*-test to determine each gene’s differential regulation and its statistical significance, as its Log_2_ fold change and p-value respectively.

The nCounter assay used in this experiment generates a count variable for each gene, not a continuous variable as found with high-throughput microarrays, and the plate assays only a small number of specifically selected prognostic genes, thus applying techniques used for microarray analysis is inappropriate. However, there is unsettled debate as how to best analyze data from expression panels^31^, we filtered, analyzed, and present our data here in three ways, from most permissive to most restrictive, to allow for the broadest interpretation of our data. First, data comparing gene expression level from each region of the brain of tumor bearing to vehicle injected mice were sorted by time of day (dark phase and light phase), analyzed by ANOVA, and then filtered using a *p* cutoff of 0.05; genes with *p* < 0.05 were considered significantly different in their expression. Post-filtering, differentially expressed genes (DEGs) from each brain region were then pooled to make an overall list of unique DEGs in the brain for each experimental group for further analyses. Next, for each brain region and experimental group, mean and standard deviation (SD) were calculated using Log_2_ Fold Change (FC) value for all genes within each experimental group. Genes with expression levels greater than or less than 2 SD from the mean of the group Log_2_ FC value were considered biologically relevant DEGs. Following FC filtering DEG lists were compiled for each experimental group as above. Finally, gene lists for each experimental group were created where both ± 2SD Log_2_ FC and *p* < 0.05 criteria were satisfied. Unique DEGs identified using each filtering approach were then analyzed using Metascape^32^.

Individual tumor volumes were calculated using the formula (width^2^ × length)/2^33^. Bilateral tumor volumes and masses were summed for each animal. Total tumor volumes and masses for body, tumor, and spleen were analyzed by two-way ANOVA. Mean differences were considered significant at *p* < 0.05.

## Results

**Figure 1:**
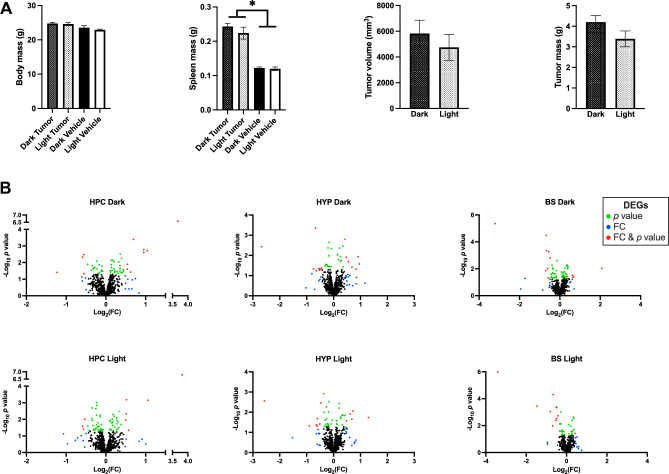
(**A**) Body mass, total bilateral tumor mass, and total bilateral tumor volume did not differ due time of day (*p* > 0.05). Mammary tumor bearing mice had larger spleens than their vehicle-injected counterparts (*p* < 0.05). (**B**) Volcano plots of gene expression comparing mammary tumor-bearing to vehicle-injected mice, by brain region and time of day. Horizontal line represents *p* = 0.05 cutoff, vertical dashed lines represent a cutoff of + /– 2 SD of Log_2_ FC value for each experimental group. *HPC* hippocampus, *HYP* hypothalamus, *BS* brain stem. Green = DEGs by *p* value, blue = DEGs by FC cutoff, red = genes by both *p* value and FC cutoff.

**Figure 2:**
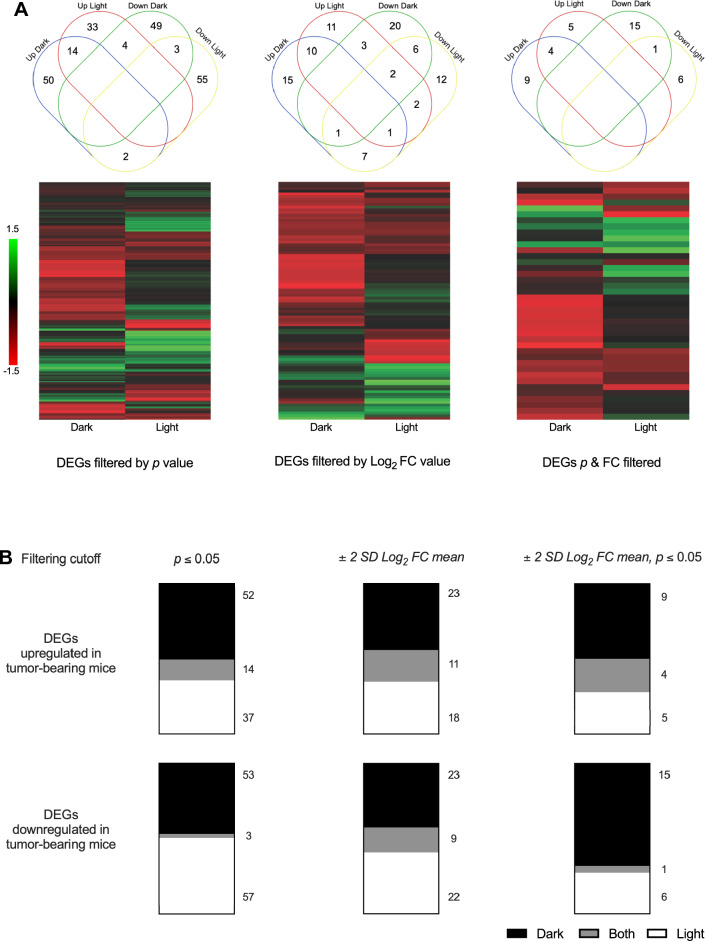
(**A**) Upper; Venn diagrams of overlap and separation between upregulated and downregulated DEGs in the brains of mammary tumor-bearing compared to vehicle-injected mice by time of day, as determined by three separate filtering methods. Lower; Expression heatmaps of all DEGs in tumor-bearing mice comparing dark to light phases. (**B**) Ratios of DEGs in the brains of mammary tumor-bearing mice compared to vehicle-injected mice by time of day, as determined by three separate filtering methods. Top: upregulated DEGs. Bottom: downregulated DEGs. Colors indicate time of day of differential expression. Numbers to the right are *n* of DEGs in each category. NB: there are few genes that are differentially expressed in the brains of mammary tumor-bearing mice independent of time of day of sampling (gray).

There were no differences in body mass (F_(1,3)_ = 0.730,* p* > 0.05), total tumor mass per animal (F_(1,3)_ = 2.646,* p* > 0.05), or total tumor volume per animal (F_(1,3)_ = 0.549,* p* > 0.05) due to time of day of collection (Fig. 1A). As expected, mammary tumor-bearing mice had larger spleens than vehicle injected mice (F_(1,3)_ = 120.349, *p* < 0.05, Fig. 1A), however, there were no differences due to time of day of collection (F_(1,3)_ = 1.014,* p* > 0.05, Fig. 1A). Relative expression level for each of the 770 genes in the assay was calculated for each of 3 brain regions at the two different times of day for sample collection, initially forming 6 groups. Within each group pre-planned comparisons of tumor-bearing to vehicle injected mice were performed, and from these comparisons each of the data filtering methods were applied to identify differentially expressed genes (Fig. 1B, Suppl Table 1). DEGs identified for each brain region were combined to form a whole brain list of unique upregulated and downregulated DEGs for further comparisons (Suppl Table 2). First gene lists were compared for overlap among regulation status and time of day. As predicted, there was little overlap between DEGs at mid-dark compared to DEGs at mid-light (Fig. 2). Indeed, regardless of DEG filtering method only 2.7–22.2% of genes were consistently differentially expressed independent of time of day in the brains of mammary tumor-bearing mice compared to vehicle injected mice (Fig. 2).

**Figure 3:**
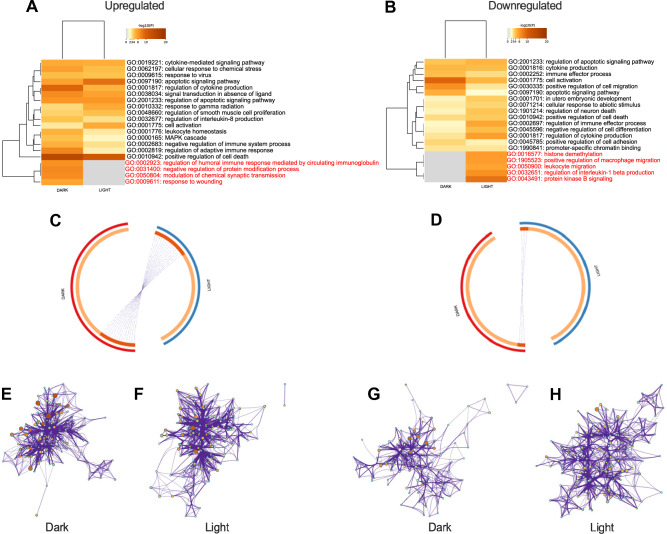
Metascape analyses of DEGs in the brains of mammary tumor-bearing mice compared to vehicle-injected mice using a *p* value cutoff of 0.05. Left column upregulated in tumor-bearing mice; right column downregulated in tumor-bearing mice. (**A**,**B**) heatmaps of GO biological processes affected by the DEGs by time of day. GO biological processes in red font are only affected during one time of day. (**C**,**D**) circos plot of overlap of individual unique genes by time of day. Outer ring red = dark, blue = light. Inner ring, dark orange represents shared genes across time of day, light orange represents unique genes to each time of day, purple lines connect individual shared genes between lists. Amount of dark red inner arc and number of purple lines indicate overlap between times of day. (**E**–**G**) GO biological enrichment networks of DEGs: (**E**) upregulated in dark, (**F**) upregulated in light, (**G**) downregulated in dark, (**G**) downregulated in light.

In the dark phase, DEGs in the brain upregulated by peripheral tumors identified by *p* < 0.05 cutoff included 20 GO biological processes, whereas sample collection in the light phase only captured differences in 16 GO processes (Fig. 3A). Similarly, downregulated DEGs sampled in the dark represented 15 GO processes, whereas collection in the light phase represented 20 GO processes (Fig. 3B). There was very little overlap in upregulated (Fig. 3C) or downregulated (Fig. 3D) individual DEGs between the dark and light phases. GO biological process enrichment networks for upregulated (Fig. 3E,F) and downregulated (Fig. 3G,H) genes visibly differ based on time of day of sample collection.

**Figure 4:**
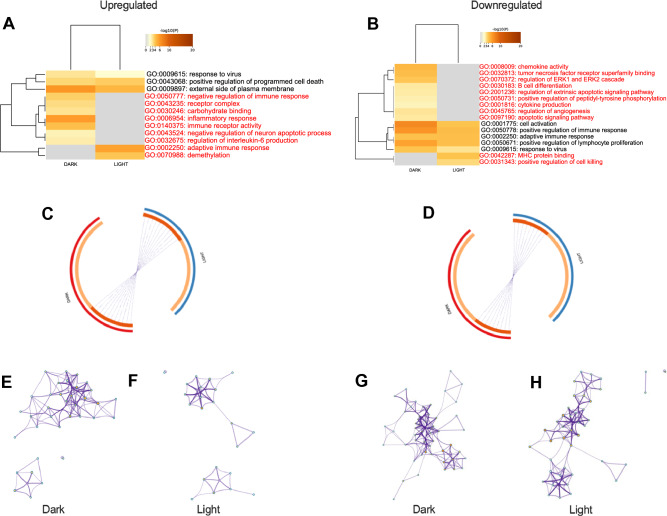
Metascape analyses of DEGs in the brains of mammary tumor-bearing mice compared to vehicle-injected mice as determined by using a cutoff of + /– 2 SD from the mean of the Log_2_ FC value. Left column upregulated in tumor-bearing mice; right column downregulated in tumor-bearing mice. (**A**,**B**) Heatmaps of GO biological processes affected by the DEGs by time of day. GO biological processes in red font are only affected during one time of day. (**C**,**D**) circos plot of overlap of individual unique genes by time of day. Outer ring red = dark, blue = light. Inner ring, dark orange represents shared genes across time of day, light orange represents unique genes to each time of day, purple lines connect individual shared genes between lists. Amount of dark red inner arc and number of purple lines indicate overlap between times of day. (**E**–**H**) GO biological enrichment networks of DEGs: (**E**) upregulated in dark, (**F**) upregulated in light, (**G**) downregulated in dark, (**H**) downregulated in light.

DEGs in the brains of tumor bearing mice identified by Log_2_ FC cutoff also differed significantly dependent on the time of day of sample collection. During the dark phase, 10 GO processes were affected by peripheral tumors, whereas only 5 were affected during the light phase (Fig. 4A). 75% of the GO processes altered by peripheral mammary tumors were time of day dependent. Similarly, almost 70% of downregulated GO processes were time of day dependent, with 14 GO processes altered in the dark phase and 7 when sampled in the light (Fig. 4B). In common with *p* value filtered data, Log_2_ FC filtered DEGs identified during the dark phase had little overlap with those from the light phase (Fig. 4C,D), and enrichment networks varied dependent on time of day of sample collection (Fig. 4E–H).

**Figure 5:**
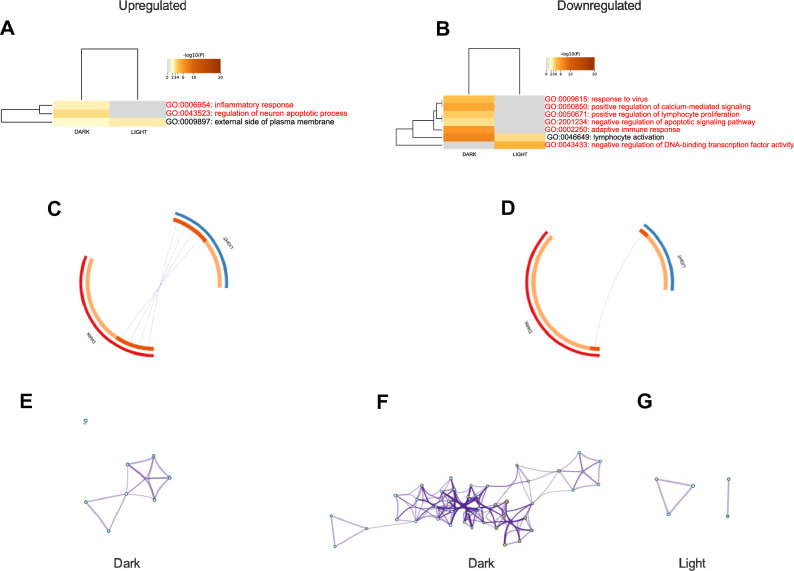
Metascape analyses of DEGs in the brains of mammary tumor-bearing mice compared to vehicle-injected mice as determined by using a cutoff of + /– 2 SD from the mean of the Log_2_ FC value, combined with a *p* value cutoff of 0.05. Left column upregulated in tumor-bearing mice; right column downregulated in tumor-bearing mice. (**A**,**B**) Heatmaps of GO biological processes affected by the DEGs by time of day. GO biological processes in red font are only affected during one time of day. (**C**,**D**) Circos plot of overlap of individual unique genes by time of day. Outer ring red = dark, blue = light. Inner ring, dark orange represents shared genes across time of day, light orange represents unique genes to each time of day, purple lines connect individual shared genes between lists. Amount of dark red inner arc and purple lines indicate overlap between times of day. (**E**–**H**); GO biological enrichment networks of DEGs: (**E**) upregulated in dark, (**F**) downregulated in dark, (**H**) downregulated in light.

Applying the most restrictive filters to identify DEGs, ± 2SD Log_2_ FC and *p* < 0.05 cutoffs, revealed a similar pattern of the strong effects of time of day of sample collection on gene expression and GO biological process networks in the brain altered by peripheral mammary tumors (Fig. 5). Indeed 2/3 of upregulated processes and over 85% of downregulated GO processes were time of day dependent (Fig. 5A–G).

## Discussion

**Figure 6:**
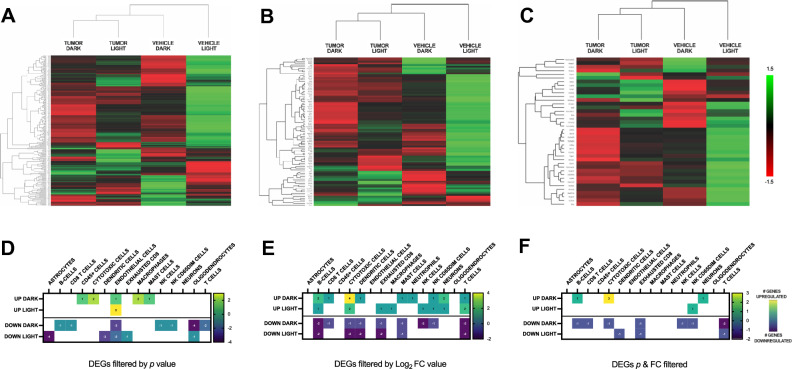
(**A**–**C**) Expression heatmaps of all DEGs in tumor-bearing and vehicle-injected mice by phase. Please see Suppl Table 1 for the lists of DEGs. (**D**–**F**) Heatmaps of DEG immune cell-type markers^30^ in tumor-bearing mice. Numbers inside blocks indicate the number of individual cell type specific DEGs; negative numbers are downregulated, positive are upregulated. (**A**,**D**) filtered by *p* value; (**B**,**E**) filtered by Log_2_ FC value; (**B**,**E**) filtered by Log_2_ FC and *p* value.

Taken together, regardless of which method of filtering is used to identify significant changes in gene expression, our data clearly reveal that time of day has a significant effect on expression patterns of immune markers in the brain driven by peripheral mammary tumors (Fig. 6). Indeed, at best only ~ 20% of DEGs are consistently up- or down-regulated independent of time of day of sampling. The implications of these time-of-day effects are obvious; significant sampling bias will be introduced into data if time of day is not considered as a critical biological variable. Although several studies have investigated the effects of breast cancer and cancer treatment on neurological and behavioral outcomes^10–12,34^, very few have considered the time of day of tissue sampling^27,35^.

Among the few papers that have explored gene expression changes in brain tissue from mice with peripheral mammary tumors based on time of day of sampling, several similarities emerge. Peripheral mammary tumors alter rhythms in hypothalamic circadian-regulated gene expression^35^. In common with our results, time of day specific effects on gene expression in the brains of tumor bearing mice have also been reported for *Cd8a, Ccl5, Cxcl10, Il1b,* and *Tlr7* (Suppl Table 1, see Table S1 in Ref.^27^). Furthermore, time of day specific dysregulation of expression patterns in the brains of tumor bearing mice is seen in genes of the Ifn, Tnf, Stat, and Il6 signaling pathways (Suppl Table 1 and Table S1 in Ref.^27^). In further support of our results, previous work examining 88 immune-response genes reported that between light and dark there was overlap in only 3 of 18 DEGs (16.7%) in the hypothalamus of peripheral mammary tumor-bearing mice (see Fig. 5E in Ref.^27^), which falls within the range of overlap we report (2.7–22.2%, Fig. 2).

Environmental light is the primary signal used by organisms to entrain their circadian rhythms to the external environment to optimize functioning of biological processes^36^. Although many preclinical research models of human health and disease are based on nocturnal rodents, the circadian rhythms of biological processes depend more upon the active and inactive phases of the organism and not necessarily whether they coincide with environmental light or dark phases^37^. Thus, the applicability of research in nocturnally-active rodents to diurnally-active human diseases states may depend more upon asking the appropriate biological questions during the same activity phase in whatever species is being studied. A more thorough approach would be to appropriately sample across the day, from both active and inactive phases, to identify the circadian profiles of the biological processes underlying whatever is being studied^2^. Only this approach will lead to a more holistic understanding of the disease or physiological process being studied.

## Conclusions

Time of day significantly alters the immune transcriptome in the brains of mice with mammary tumors when compared to vehicle-injected mice. Sampling bias by measuring physiological variables or collecting tissues and not controlling for time of day will introduce experimental variability and significantly alter interpretation of experimental outcomes based on the number of biological processes that vary based on time of day.

### Supplementary Information

Supplementary Table 1.

Supplementary Table 2.

Supplementary Legends.

## Data Availability

The datasets used and/or analyzed during the current study have been deposited in NCBI's Gene Expression Omnibus and are accessible through GEO Series accession number GSE249022 (https://www.ncbi.nlm.nih.gov/geo/query/acc.cgi?acc = GSE249022).

## Abbreviations

LD: Light:Dark
ZT: Zeitgeber time
HYP: Hypothalamus
HPC: The hippocampus
BS: Brainstem
DEGs: Differentially expressed genes
SD: Standard deviation
FC: Fold change
