# Supplementary material for: Time of day bias for biological sampling in studies of mammary cancer
Source: Sci Rep. 2024 Jan 8;14:848. doi: 10.1038/s41598-023-50785-y (PMC10774401; doi:10.1038/s41598-023-50785-y)
Supplement: Supplementary file 3 — Supplementary Legends. [file 41598_2023_50785_MOESM3_ESM.docx]

Table 1 List of all genes, the respective analyses, and NCBI classification data. Abbreviations: BS - brain stem, Hippo - hippocampus, Hy - hypothalamus,

Table 2 List of all DEGs identified by each filtering method, sorted by phase and direction of regulation. Genes with confirmed cell type marker characteristics 30 are labeled by cell type.
